# Distinct Recycling of Active and Inactive β1 Integrins

**DOI:** 10.1111/j.1600-0854.2012.01327.x

**Published:** 2012-01-31

**Authors:** Antti Arjonen, Jonna Alanko, Stefan Veltel, Johanna Ivaska

**Affiliations:** 1 Medical Biotechnology VTT Technical Research Centre of FinlandTurku 20521 Finland; 2 Centre for Biotechnology University of TurkuTurku 20520 Finland; 3 Department of Biochemistry and Food Chemistry University of TurkuTurku 20520 Finland

**Keywords:** conformation, endocytosis, integrin, recycling, trafficking

## Abstract

Integrin trafficking plays an important role in cellular motility and cytokinesis. Integrins undergo constant endo/exocytic shuttling to facilitate the dynamic regulation of cell adhesion. Integrin activity toward the components of the extracellular matrix is regulated by the ability of these receptors to switch between active and inactive conformations. Several cellular signalling pathways have been described in the regulation of integrin traffic under different conditions. However, the interrelationship between integrin activity conformations and their endocytic fate have remained incompletely understood. Here, we have investigated the endocytic trafficking of active and inactive β1 integrins in cancer cells. Both conformers are endocytosed in a clathrin- and dynamin-dependent manner. The net endocytosis rate of the active β1 integrins is higher, whereas endocytosis of the inactive β1 integrin is counteracted by rapid recycling back to the plasma membrane via an ARF 6- and early endosome antigen 1-positive compartment in an Rab 4a- and actin-dependent manner. Owing to these distinct trafficking routes, the two receptor pools display divergent subcellular localization. At steady state, the inactive β1 integrin is mainly on the plasma membrane, whereas the active receptor is predominantly intracellular. These data provide new insights into the endocytic traffic of integrins and imply the possibility of a previously unappreciated crosstalk between pathways regulating integrin activity and traffic.

Cell migration is critically co-ordinated by trafficking of plasma membrane receptors 1. Integrins are a large family of heterodimeric cell surface adhesion receptors consisting of 24 non-covalently associated α and β subunits with overlapping substrate binding 2. The binding of extracellular matrix ligands to integrin heterodimers induces a conformational change in the extracellular β subunit, followed by a separation of the α/β cytoplasmic tails 3,4. The tail separation creates space for binding of many cytoskeletal proteins and adaptor proteins (APs), leading to a focal adhesion complex build-up in the cytosolic interface 5. Integrin-containing adhesions allow cells to exert adhesion forces between different parts of the cell body which is a prerequisite for cell shape changes and cell motility 6,7.

In migrating cells, membrane trafficking is orchestrated with the actin cytoskeleton to promote the formation of new adhesions and lamellipodial protrusions on the cell. Endocytosis and recycling of integrins, growth factor receptors and lipids is necessary for maintaining cell polarization and to co-ordinate the formation and release of cell-substrate attachments^1^,^8^. Integrins are known to undergo constant endo/exocytic traffic in adherent cells 9. The endocytosed integrins are predominantly recycled back to the membrane. In comparison to receptor traffic, the degradation of integrins is slow. The pool of cell surface α5β1 integrins is endocytosed and recycled once every 30 min back to plasma membrane^10^,^11^, whereas the half-life of integrin α5β1 in lysosomal degradation is 18 h 12. This highlights the importance of integrin traffic in cell migration and in the maintenance of an appropriate balance between internalized and cell surface integrins in cells.

The endocytosis of integrin heterodimers occurs by clathrin-dependent and clathrin-independent mechanisms. Ligand occupied α2β1 is internalized in a clathrin/Rab21-dependent manner 13,14, whereas α5β1, αVβ3 and virus/antibody clustered α2β1 have been shown to associate with caveolin-1 15. However, the same heterodimer may use multiple pathways for endocytosis. For example, α5β1 integrin endocytosis is caveolin dependent during fibronectin assembly 16, but clathrin-dependent endocytosis of α5β1 has been described in migrating cells as well as during cytokinesis 17,18.

Thus far, four integrin recycling pathways have been described: The Rab4-dependent short-loop, Rab11-dependent long-loop, stimulation-dependent ARF6 pathway and tubular actin-dependent recycling endosomes. Integrin αVβ3 has been shown to cycle via the short-loop pathway, whereas β1 integrins have been described to traffic via the long-loop route 10,19,20. The actin-dependent recycling pathway has been described for β1 integrins and for β2 adrenergic receptor^21^,^22^.

In addition to integrin trafficking, integrin activity has a clear impact on cell motility as well. Extracellular matrix (ECM) ligand concentrations, integrin expression levels and integrin–ECM binding affinities all contribute to cell migration 23. The activity of integrins is based on the conformation of the heterodimeric receptor. The current model suggests an equilibrium among (i) an inactive, bent conformation with a closed headpiece, (ii) a primed, extended conformation with a closed headpiece and (iii) an active, extended conformation with a ligand bound open headpiece 3. It has been suggested that β1 integrins are mainly in an inactive conformation at the cell surface 24. Integrin activation occurs via inside-out or outside-in signalling events. These trigger intracellular conformational changes and the separation of the cytoplasmic tails. The conformational change from a bent to an extended conformation increases integrin-ligand affinity 25,26. The inside-out activation of integrins is induced by binding of cytoplasmic adaptors like talin-1/2 and kindlin-1/2/3 to the β1 integrin cytoplasmic tail. Integrin activity is inhibited by binding of Shank-associated RH domain-interacting protein (SHARPIN) to the α-cytoplasmic tail 27.

Individual studies have indicated differences in the endocytosis of active and inactive integrin β1 conformations. Focal adhesions are known to contain clustered, talin-associated and ECM-bound integrins 28. The disassembly of focal adhesions involves clathrin/Dab2/Arh-dependent endocytosis of ECM-bound α5β1 integrins 29,30. Thus, this pathway would be predicted to target activated integrins for uptake. However, clathrin adaptor Dab2-dependent endocytosis has also been shown to target free non-occupied α1β1, α2β1 and α3β1 integrins 31. Another clathrin adaptor, Numb, is involved in focal adhesion disassembly and in β1 integrin endocytosis, but the activity status of these integrins remains unclear 17. Neuropilin-1 has been linked to endocytosis of active α5β1 integrin in endothelial cells 32, and endocytosis of inactive α2β1 integrins is induced during echovirus 1 internalization into cells 33. However, because of different cell lines and experimental methodology used, these data are difficult to compare and to the best of our knowledge, a comprehensive analysis of the trafficking of inactive and active pools of β1 integrins is missing.

Here, we have studied the endocytic trafficking of active and inactive β1 integrins. Using antibody-based detection, we demonstrate distinct trafficking kinetics for active and inactive β1 integrins. The net endocytosis rate of active β1 integrin is higher, whereas the inactive β1 integrin endocytosis is increasingly shifted toward Rab4a and actin-dependent recycling back to the plasma membrane protrusions.

## Results

### Active β1 integrins are predominantly cytoplasmic, whereas inactive β1 integrins localize to the plasma membrane and protrusions

**Figure 1:**
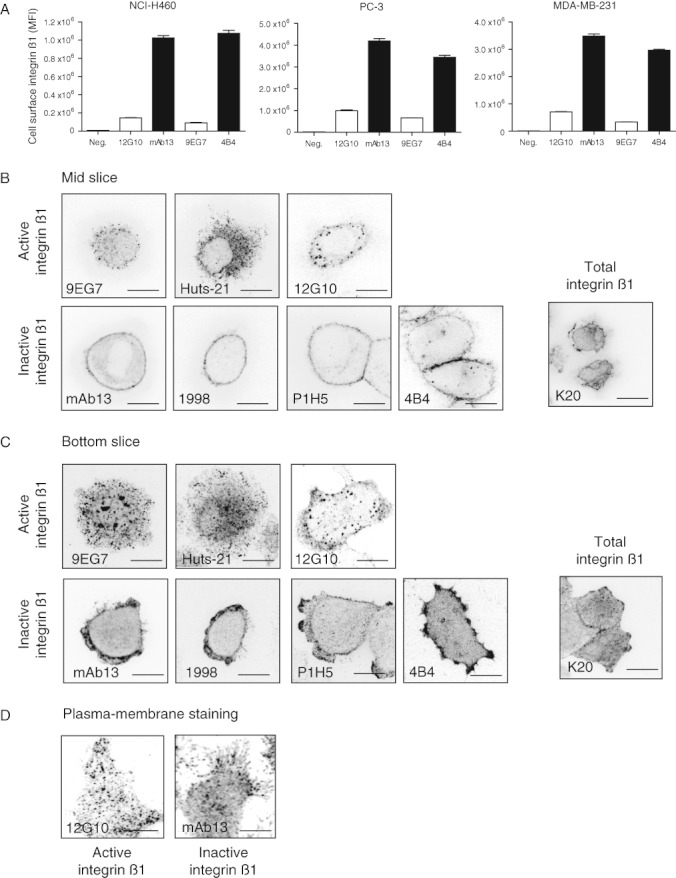
Active β1 integrins are predominantly cytoplasmic, whereas inactive β1 integrins localize to the plasma membrane and protrusions. A) NCI-H 460, PC-3 and MDA-MB -231 cells were harvested with HyQ tase and cell surface β1 integrin expression was measured with flow cytometry. Indicated antibodies against active (12 G1 0 and 9 EG 7, open bars) and inactive (mAb 13 and 4 B 4, black bars) β1 integrin were used. Columns show mean fluorescent intensities ( MFI ) and mean + standard error of the mean of three independent experiments. B and C) NCI-H 460 cells were grown under regular cell culture conditions. The cells were fixed, permeabilized and stained as indicated. B) Confocal mid-sections of representative cells stained with antibodies against active β1 integrin (9 EG 7, Huts -21 and 12 G 10), inactive β1 integrin (mAb 13, 1998, P 1 H 5 and 4 B 4) and total β1 integrin ( K 20) are shown. C) Confocal bottom slice sections of representative cells stained against β1 integrins as above are shown. D) Total internal reflection fluorescence microscopy images of NCI-H 460 cells stained against active β1 integrin (12 G 10) and inactive β1 integrin ( mAb 13). Scale bar 10 µm.

Monoclonal anti-integrin β1 antibodies have been well characterized, and their ability to detect conformation-dependent epitopes has been defined 34. We measured the levels of active and inactive β1 integrin expressions on cell surface using flow cytometry and epitope-specific antibodies. In three independent cancer cell lines (NCI-H460, PC-3 and MDA-MB-231), the cell surface β1 integrins were found to be mainly in the inactive conformation (mAb13 and 4B4), whereas approximately only 20% of cell surface β1 integrins were in the active conformation (12G10 and 9EG7) (Figure 1A). This is in line with a previous report 24. Interestingly, the immunofluorescence staining of fixed, unstimulated NCI-H460 lung carcinoma cells shows distinct localizations of active and inactive β1 integrins. Confocal mid-sections of cells reveal that active β1 integrins (detected with 9EG7, Huts-21 and 12G10 mAbs) are more intracellular and show endosomal staining inside the cell body (Figure 1B). Conversely, the inactive β1 integrins (detected with mAb13, 1998, P1H5 and 4B4 mAbs) localize to the plasma membrane and to protrusions (Figure 1B,C). The total β1 integrin pool stained with K20 antibody shows intracellular and plasma membrane staining. At the bottom of cells, the active β1 integrin shows punctate- and focal contact-like staining throughout the basal side of the cell (Figure 1C). The inactive β1 integrin is highly enriched in protrusions at the cell edges (Figure 1C). Furthermore, total internal reflection fluorescence microscopy shows that the active β1 integrins are more clustered at the plasma membrane compared to the inactive conformation (Figure 1D). On the basis of these observations, we hypothesized that the endosomal trafficking of integrins would be different for the active and the inactive receptors.

### The active β1 integrin is internalized more efficiently than the inactive conformation

**Figure 2:**
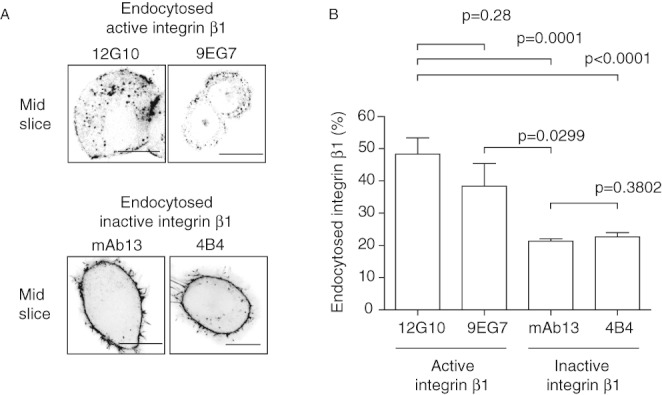
The active β1 integrin is internalized more efficiently than the inactive conformation. A) NCI-H 460 cells were grown under regular cell culture conditions. Cells were lifted on ice and stained for 1 h with the indicated monoclonal anti-β1 integrin antibodies. Cells were lifted back to +37°C and allowed to endocytose the primary antibodies for 30 min. Cells were fixed, permeabilized and stained with secondary antibodies. The mid-section of a confocal stack is shown. B) The intensity of endocytosed integrin was quantified from the confocal images (*n* = 10–16) using a region of interest (ROI) inside the cell and normalized against the total staining of the cell. Scale bar 10 µm. Columns show mean + standard error of the mean. p V alues are calculated using M ann– W hitney test.

The differences in the cell surface β1 integrin levels and in the staining patterns of fixed cells at steady state would implicate preferred endocytosis of the active integrins. Thus, we labelled cell surface β1 integrins in living cells with monoclonal primary antibodies against active and inactive conformations. A previous study using antibodies to study integrin endocytosis obtained identical trafficking results with immunoglobulin G (IgG) molecules and monovalent Fab-fragments, suggesting that the possible clustering effect of IgG-molecules has no significant impact on integrin traffic 21. We followed the endocytosis of antibody-labelled cell surface integrins in serum-containing medium without additional stimulation of cells. The primary antibody chase showed significantly more endocytosis of active β1 integrin recognizing antibodies compared to the inactive β1 integrins (Figure 2A,B).

### Antibody- and quenching-based method allows direct measurement of integrin trafficking

**Figure 3:**
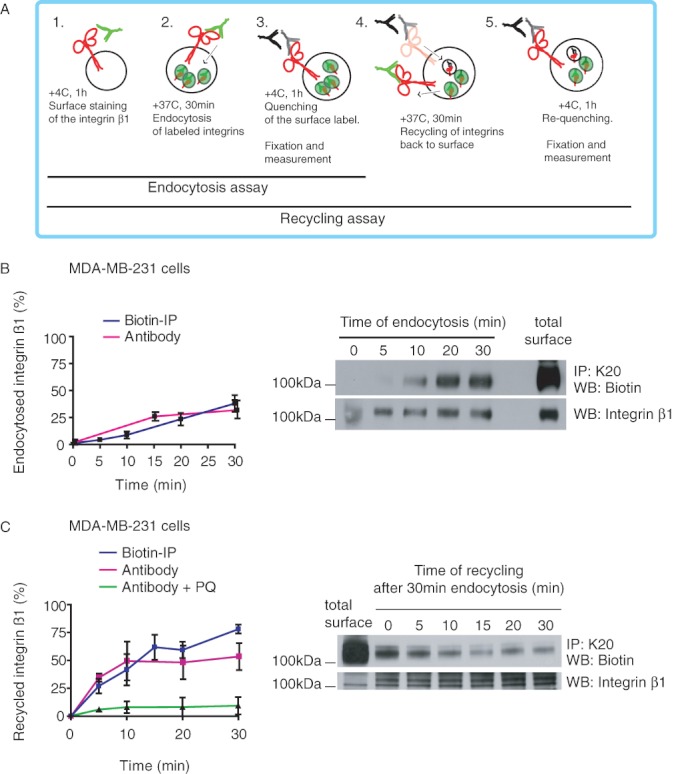
Antibody- and quenching-based method allows direct measurement of integrin trafficking. A) Outline of the antibody-based integrin trafficking assay for measuring endocytosis and recycling. B) Comparison of β1 integrin endocytosis in MDA-MB -231 cells with antibody-based and biotin– IP -based assays using total β1 integrin K 20 antibody. Graph shows mean ± standard error of the mean ( SEM ) of three independent experiments. Total surface and endocytosed biotin-labelled β1 integrin are shown in the western blot. C) Comparison of β1 integrin recycling in MDA-MB -231 cells with antibody-based and biotin– IP -based assays using total β1 integrin K 20 antibody. PQ was used to block the endosomal recycling of integrins (green line). Graph shows mean ± SEM of three independent experiments. Total surface, total endocytosed (30 min) and recycled biotin-labelled β1 integrins are shown in the western blot.

To better analyse β1 integrin endocytic trafficking, we developed a fluorescence-based assay to measure the endocytosis and recycling rates of β1 integrins(Figure 3A). The method is based on quenching (95% efficacy) of the fluorescent signal of Alexa Fluor 488-conjugated anti-β1 integrin antibodies on the plasma membrane using a specific anti-Alexa Fluor 488 antibody (Figure S1A). The quenching antibody has previously been successfully used to study the endocytosis of CD8 35. We tested our method using a fluorescent multiwell plate-reader to analyse integrin endocytosis in adherent cells and flow cytometry to analyse cells in suspension under similar conditions. Endocytosis is temperature dependent and clearly more efficient in adherent cells (Figure S1B). To validate the results of the antibody-based integrin trafficking method, we reproduced the experiments using previously published cell surface biotinylation, immunoprecipitation and western blotting-based assays for comparison (10). The antibody-based assay showed similar kinetics and end-point rates of β1 integrin endocytosis and recycling as the biotin-immunoprecipitation (IP)-based assay. Approximately 45% of the cell surface β1 integrins were endocytosed in 30 min (Figure 3B) in MDA-MB-231 cells. PC-3 cells showed similar kinetics of β1 integrin endocytosis with both assays (Figure S2A). The recycling of β1 integrins was approximately 70% in 30 min with the biotin–IP assay. A slightly lower 30-min end-point rate of β1 integrin endocytosis was obtained with the antibody-based assay, but the kinetics were nearly the same (Figure 3C). Blocking of endosomal recycling with the anti-malaria drug Primaquine (PQ), an established inhibitor of integrin recycling 36,37, showed a clear inhibitory effect in our antibody-based assay (Figure 3C). Similar levels of β1 integrin endocytosis and recycling were also measured with ELISA-based detection in biotin–IP assay (Figure S2B,C).

There were differences in the sensitivity, linearity and the total signals depending of the detection method. However, none of the methods produced significantly discrepant results or showed systematically lower or higher kinetics or end-point rates. Therefore, we conclude that our antibody-based assay is a valid method to detect integrin traffic in cells.

### Cell surface bound monoclonal anti-β1 integrin antibodies do not significantly trigger downstream signalling

The labelling of the cell surface with activating anti-β1 integrin antibodies could in theory induce cell signalling and thus lead to increased endocytosis. In order to evaluate the effects of antibody labelling in live cells, we tested whether the labelling regime used in the trafficking assays would influence downstream integrin signalling. Addition of anti-β1 integrin antibodies (9EG7, 12G10 and mAb13) with or without secondary antibody clustering or fibronectin ligand to the cell surface did not significantly influence the phosphorylation of focal adhesion kinase 1 and p42/44 (Figure S3). On the basis of these data, we conclude that integrin labelling used here and in previous work published by us and others does not significantly trigger integrin signalling in cells.

### Active β1 integrin has higher net endocytosis rate and co-traffics with ligand

**Figure 4:**
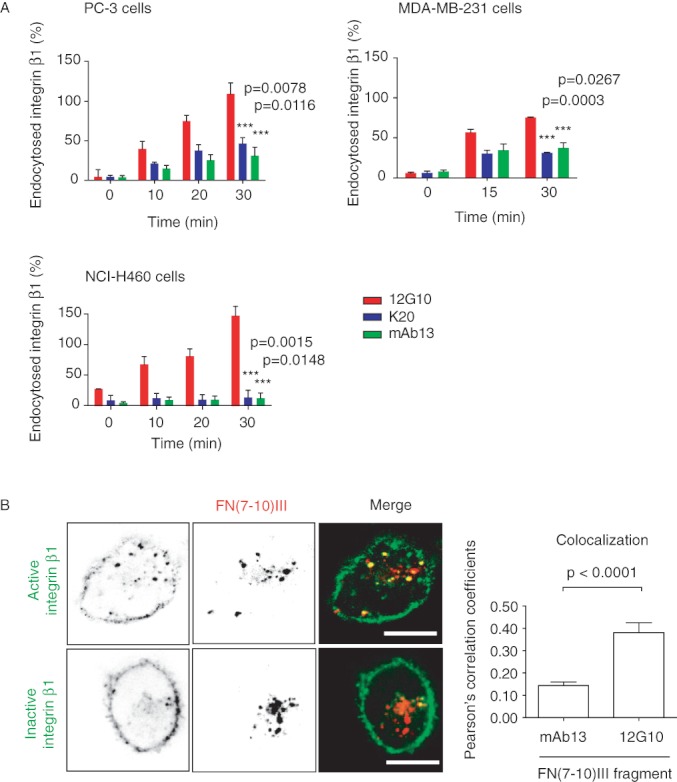
Active β1 integrin has a higher net endocytosis rate and co-traffics with ligand. A) PC -3, MDA-MB -231 and NCI-H 460 cells were labelled with monoclonal antibodies against active, inactive and total β1 integrin (12 G 10, mAb 13 and K 20, respectively). The antibody-based integrin endocytosis assay was used to measure integrin β1 endocytosis over time. Columns show mean + standard error of the mean ( SEM ) of three independent experiments. p V alues are calculated using M ann– W hitney test. B) MDA-MB -231 cells were surface labelled for 1 h on ice with A lexa F luor 488-labelled fibronectin fragment FN (7-10) and allowed to internalize the ligand for 15 min at 37°C. Cells were then surface labelled with A lexa F luor 647-labelled anti-β1 integrin antibodies against active and inactive conformations (12 G1 0 and mAb 13) for 1 h on ice and allowed to internalize the antibodies for 30 min at 37°C. Mid-slice confocal images are shown. Columns show mean + SEM of colocalization over the whole image as PC coefficients (*n* = 10). p V alues are calculated using M ann– W hitney test.

Having validated the antibody-based traffic assay, we compared the endocytic rates of different β1 integrin cell surface populations using antibody chase as a function of time. In line with the previous primary antibody-chase experiments, both the active and inactive β1 integrin conformations were endocytosed in a time-dependent manner (Figure 4A). We verified the results using three cancer cell lines (PC-3, MDA-MB-231 and NCI-H460, respectively). The active β1 integrin showed significantly higher net endocytosis rates when compared to the inactive conformer or the total pool of all β1 integrins in all three cell lines.

Fibronectin has been detected in integrin-containing endosomes^13^,^38^. In addition, ubiquitination followed by FN-ligand binding targets a fraction of α5β1 integrin to specific lysosomal compartments 12. To visualize active and inactive β1 integrin trafficking, we chased the antibodies together with a labelled fibronectin fragment FN(7-10)III. As expected, the co-endocytosis and overlap of active β1 integrin and the FN-fragment were higher (Pearson's correlation, PC = 0.4) than with the inactive β1 integrin (PC = 0.15). The inactive β1 integrin was not endocytosed together with ligand-engaged integrins (Figure 4B). In summary, these data suggest distinct trafficking of active and inactive β1 integrins. The active integrin is co-endocytosed and co-trafficked with the ligand. In contrast, the unoccupied inactive integrin is not significantly present in ligand-containing structures and shows reduced net endocytosis.

### Trafficking routes of active and inactive β1 integrins overlap in early endosomes but only active β1 localizes to a Rab 7-positive compartment

**Figure 5:**
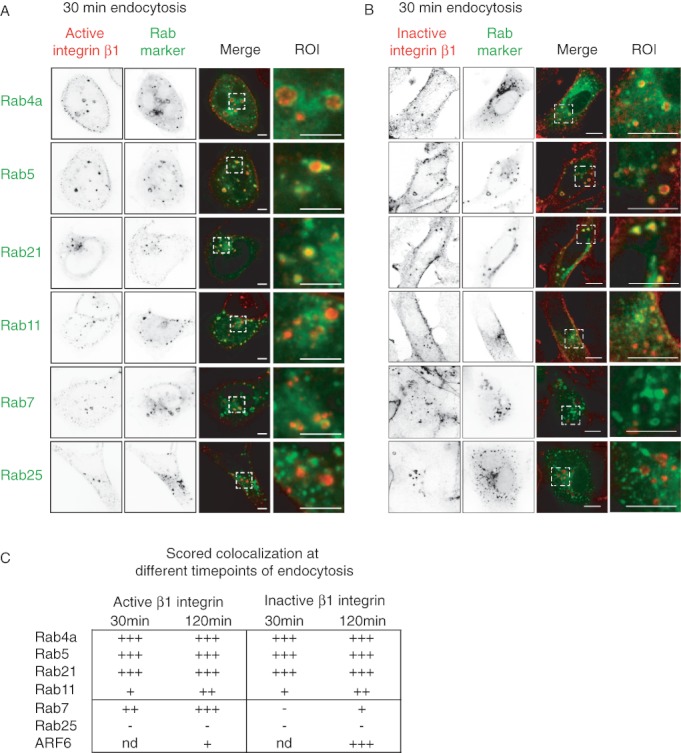
The endosomal trafficking pathway of active and inactive β1 integrins. MDA-MB -231 cells were transfected with EGFP -tagged small Rab GTPases and surface stained with antibodies against active (12 G1 0) (A) or inactive ( mAb 13) (B) β1 integrins. Integrins were allowed to endocytose for 30 min and cells were fixed, counterstained and analysed under confocal microscope. Mid-slices and ROI are shown. Scale bar 10 µm. C) Colocalization of β1 integrins and different small Rab - GTPases was scored by visual comparison. Three plus indicates good and clear colocalization in endosomes, whereas single plus only partial colocalization. Minus indicates no colocalization. Nd stands for not determined.

To identify the trafficking pathways of active and inactive β1 integrins, we followed antibody-labelled integrins in cells transfected with small Rab-GTPases, which are known to localize to specific endosomal compartments 39. Active and inactive β1 integrins were clearly found in Rab5-, Rab4a- and Rab21-positive compartments 30 min after endocytosis (Figure 5A,B). However, only the active β1 integrin was detected in Rab7-positive compartments 30 min after endocytosis (Figure 5B, scored table in Figure 5C). One hundred and twenty minutes after endocytosis, Rab11 perinuclear recycling compartment (PNRC) was also positive for both conformations, whereas Rab25 showed no clear colocalization in either time-points (Figure S4B). These observations indicate that ligand occupied β1 integrins may be *en route* to lysosomal degradation as has been suggested before 12. The observed difference does not directly explain the higher net endocytic rate of active β1 integrin as both conformations colocalize to the same extent with early endosomal markers (Rab5 and Rab21) and fast-loop recycling endosome marker (Rab4a).

### The endocytosis of active and inactive β1 integrins is dynamin and clathrin dependent

**Figure 6:**
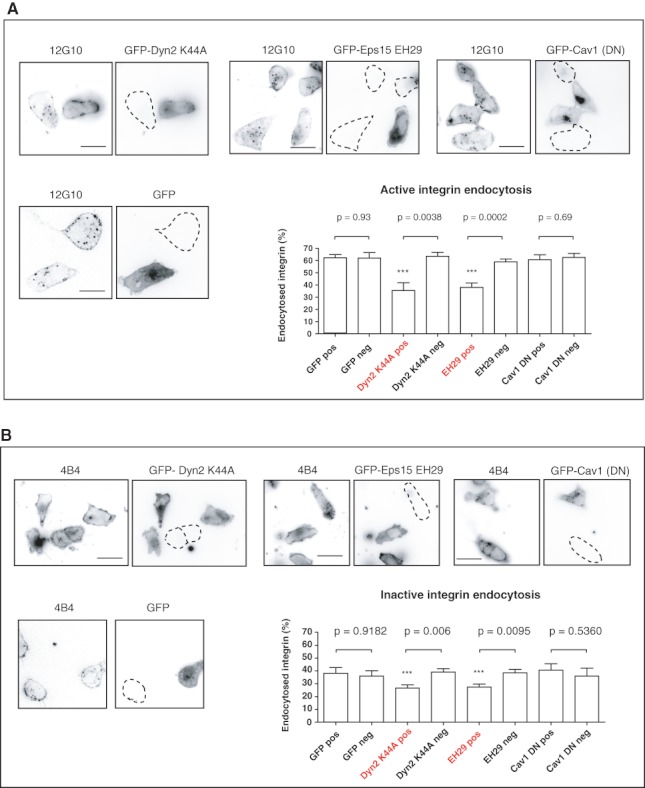
The endocytosis of active and inactive β1 integrins is dynamin and clathrin dependent. MDA-MB-231 cells were transfected with GFP-tagged dominant-negative dynamin-2 (K44A), dominant-negative Eps15 (EH29), dominant-negative caveolin-1 (EGFP-caveolin-1) and EGFP alone. A) Active β1 integrins (12G10) were surface labelled for 1 h on ice, and cells were allowed to endocytose the antibodies for 30 min at 37°C. B) Inactive β1 integrins (mAb13) were surface labelled for 1 h on ice, and the cells were allowed to endocytose the antibodies for 30 min at 37°C. In both (A) and (B), the amount of endocytosed integrin was quantified from confocal mid-sections and normalized to the total staining of the cell. The striated lines mark for untransfected cells. Columns show mean + standard error of the mean of GFP-positive and GFP-negative cells. p Values are calculated using Mann–Whitney test (*n* = 10–17). Scale bar 10 µm.

Distinct endocytosis routes could underlie the differences in the observed trafficking of active and inactive β1 integrins. We transfected MDA-MB-231 cells with dynamin-2 K44A, Eps15 EH29 or dominant-negative caveolin-1 to pertubate the canonical endocytic routes. Dynamin-2 mutant K44A blocks the dynamin-dependent abscission of endocytic vesicles 40. Eps15 lacking the EH domains disturbs the AP2–clathrin complex formation and thus blocks clathrin-mediated endocytosis 41. N-terminally enhanced green fluorescent protein (EGFP)-tagged caveolin-1 has been shown to function as a dominant negative (DN) inhibitor of SV40 internalization into cells 42. Antibody chase against active and inactive β1 integrins in the transfected cells showed that dynamin-2 K44A and Eps15 EH29 inhibited the endocytosis of both conformations, whereas the EGFP-caveolin-1 (DN) had no effect on the endocytosis of either conformation (Figure 6). We used transferrin endocytosis (known to be clathrin and dynamin dependent 43) as a positive control and to validate the functionality of the system (Figure S5A). In line with the clathrin dependency of integrin endocytosis, clathrin colocalized with both chased active and inactive β1 integrin in endosomal puncta, whereas caveolin-1 did not (Figure S5B,C). These results indicate that the initial steps of endocytosis are shared by the two receptor conformations.

### Inhibition of recycling increases the amount of endocytosed inactive β1 integrins in endosomes

**Figure 7:**
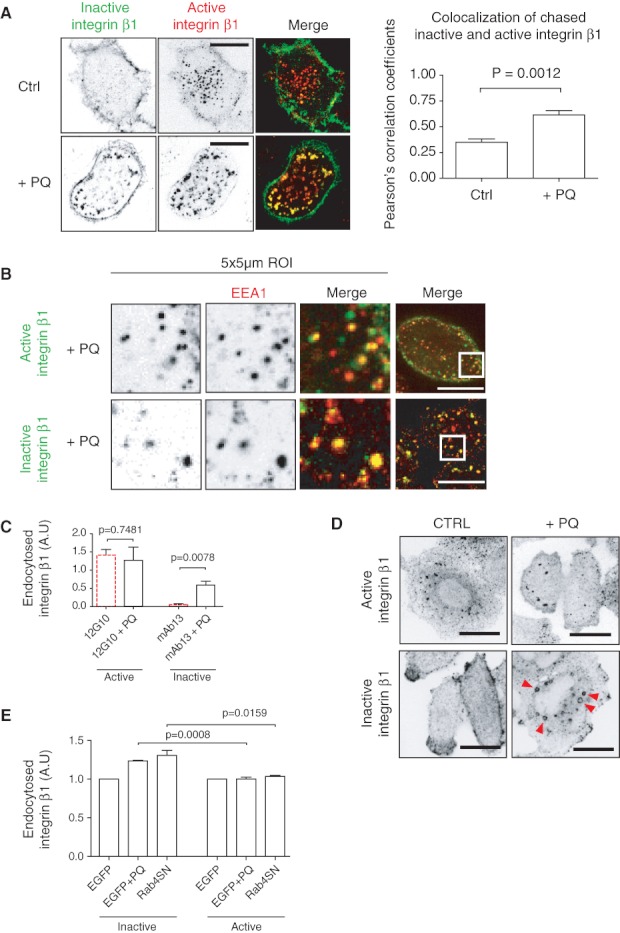
Inhibition of recycling increases the amount of endocytosed inactive β1 integrins. A) NCI-H460 cells were double labelled for 1 h on ice with β1 integrin antibodies against active (12G10) and inactive (mAb13) conformations. To block the recycling, 0.5 m m PQ was added during the 30-min endocytosis at +37°C. Cells were fixed and imaged with confocal microscope. Scale bar 10 µm. Columns show mean + standard error of the mean (SEM) of PC coefficients of the colocalization over the whole image between active and inactive β1 integrins (*n* = 10). p Values are calculated using Mann–Whitney test. B) NCI-H460 cells treated with 0.5 m m PQ were allowed to endocytose antibodies against active (12G10) and inactive (mAb13) β1 integrins. Cells were fixed, permeabilized and stained against early endosome marker EEA1. Scale bar 10 µm. C) Results of antibody-based endocytosis assay using fluorescent plate-reader. NCI-H460 cells were allowed to endocytose active (12G10) and inactive (mAb13) β1 integrin with and without 0.5 m m PQ for 30 min at 37°C. The graphs show mean + SEM of three independent experiments. p Values are calculated using Mann–Whitney test. D) NCI-H460 cells were treated with 0.5 m m PQ for 30 min at 37°C, fixed and stained against active (12G10) and inactive (mAb13) integrin β1. Arrowheads point to endosomes containing inactive β1 integrin. E) MDA-MB-231 cells were transfected with EGFP-alone or EGFP-Rab4a-S22N. Cells were harvested with HyQtase and surface stained against inactive (mAb13) or active (12G10) β1 integrins. Integrins were allowed to endocytose for 60 min, and the level of cell surface β1 integrin was analysed from non-treated, PQ-treated and EGFP-Rab4a-S22N positive cells before and after endocytosis. Columns show level of endocytosed β1 integrin normalized to EGFP-control, mean + SEM of three independent experiments. p Values are calculated using unpaired *t*-test.

As both conformations are dependent on dynamin and clathrin for endocytosis, we considered the possibility that distinct recycling rates of the active and inactive integrins would underlie the observed higher net endocytic rates of the active β1 integrin. To test this, we labelled the cell surface simultaneously with antibodies against active and inactive β1 integrins. After a 30-min antibody chase, the cells were fixed, counterstained and analysed. Again, the staining of the inactive β1 integrin was mostly seen at the plasma membrane, whereas the active β1 integrin was more intracellular (Figure 7A). Interestingly, the overlap between endocytosed active and inactive β1 integrin increased significantly (from 0.32 to 0.6 PC) after inhibiting the recycling from endosomes to the plasma membrane with PQ. The increased colocalization was detected in early endosome antigen 1 (EEA1)-positive compartments close to the plasma membrane (Figure 7B).

Next, we compared the rates of active and inactive β1 integrin endocytosis at the 30-min time-point. Blocking of the recycling increased the net endocytosis of the inactive β1 integrin significantly, whereas the net endocytosis of the active β1 integrin was not markedly altered (Figure 7C). This was reflected in the appearance of the endocytosed inactive β1 integrin in round endosomal looking compartments in the presence of PQ (Figure 7D, bottom row). These structures were also detected in cells stained after fixation with different inactive β1 integrin antibodies (mAb13, 1998, P1H5 and 4B4), indicating that the formation of these endosomes was not due to the antibody chase (Figure S6). The subcellular localization of the endocytosed active β1 integrin was slightly changed following PQ treatment, indicated by trapping to early endosomes (Figure 7A, bottom row), but the amount of endocytosed active β1 integrin was not markedly changed (Figure 7C,D, top row). Unlike the inactive receptor, active β1 integrin did not form swollen endosomes in the presence of PQ.

### Inactive β1 integrin undergoes Rab 4-dependent recycling

In fibroblasts, αVβ3 integrin has been shown to recycle back to the plasma membrane in a Rab4a-dependent manner followed by growth factor stimulation 10. Because in our study, both β1 integrin conformations showed clear colocalization with Rab4a (Figure 5C), we tested whether dominant-negative Rab4a (Rab4a-S22N) would affect β1 integrin recycling. Expression of EGFP-Rab4a-S22N increased the amount of endocytosed inactive β1 integrins to a similar extent as PQ treatment (Figure 7E). Rab4a-S22N had no effect on active β1 integrin. These data suggest the inactive, but not the active, β1 integrin is actively recycled back to plasma membrane via a Rab4-dependent fast-loop pathway and thus the net endocytic rate of the inactive β1 integrin is lower compared to the slower recycling active β1.

### Inactive β1 integrin recycling is dependent on actin

**Figure 8:**
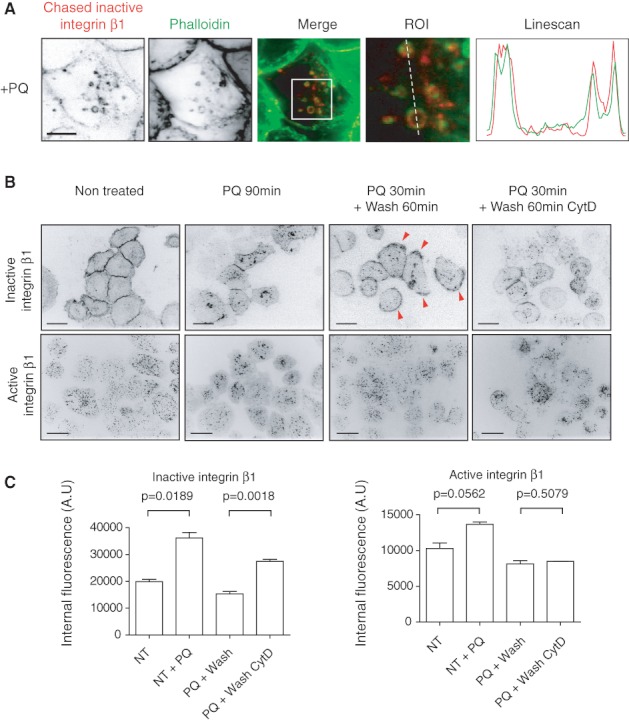
Inactive β1 integrin recycling is dependent on actin. A) NCI-H 460 cells were labelled for 1 h on ice and allowed to endocytose inactive β1 integrin ( mAb 13, A lexa F luor 647-labelled) for 30 min at 37°C with 0.5 m m PQ. Cells were fixed and stained with A lexa F luor 488 P halloidin to visualize F -actin. A line scan and ROI are shown on the right. Scale bar 10 µm. B) NCI-H 460 cells were surface labelled against inactive ( mAb 13) or active (12 G1 0) β1 integrin for 1 h on ice and incubated at 37°C with growth medium (non-treated) for 90 min, with 0.5 m m PQ for 90 min, with 0.5 m m PQ for 30 min followed by a 60-min medium wash, with 0.5 m m PQ for 30 min followed by a 60-min wash with medium containing 20 m m CytD. Mid-slice confocal images are shown. Arrowheads point to membrane-relocalized inactive β1 integrin. Scale bar 10 µm. C) NCI-H 460 cells were surface labelled against inactive ( mAb 13) or active (12 G1 0) β1 integrin 60 min on ice and incubated at 37°C with growth medium (non-treated) for 60 min, with 0.5 m m PQ for 60 min, with 0.5 m m PQ for 30 min followed by a 30-min medium wash, with 0.5 m m PQ for 30 min followed by a 30 min wash with medium containing 20 m m CytD. Cells were lifted on ice and cell surface fluorescence was quenched. The level of internal β1 integrin was measured using automated fluorescent microscope ScanR. Columns show mean + standard error of the mean of 6000–8000 cells. P V alues are calculated using unpaired *t*-test.

It has been previously shown that rapid β1 integrin recycling is an F-actin-dependent process 20,21. In these studies, actin polymerization has been inhibited with the drug Cytochalasin D (CytD) or by overexpressing point mutated ARF6 (Q37E/S38I) which also inhibits the recycling of the β1 integrin 20,21. We found that PQ-induced inactive β1 integrin endosomes significantly overlapped with F-actin staining (Figure 8A), in line with the notion of F-actin-dependent recycling endosomes. Next, we analysed the functional relationship between inactive β1 integrin recycling and actin polymerization. We studied the effect of CytD in the localization of endocytosed β1 integrin after PQ washout (Figure 8B). As shown earlier, in non-treated cells, the inactive β1 integrin localized predominantly to the plasma membrane, whereas the active β1 integrin was mainly endosomally localized. PQ treatment significantly induced accumulation of the inactive β1 integrin inside the cells (Figure 8B, second column, top). After 60-min PQ washout, the inactive β1 integrin was relocalized to plasma membrane protrusions (Figure 8B, third column, top, arrowheads), whereas no apparent changes were detected in the localization of the active β1 integrin. Inhibition of F-actin polymerization with CytD during PQ washout blocked the recycling of inactive β1 integrin to the plasma membrane (Figure 8B, last column, top). No obvious redistribution of the active β1 integrin was observed after PQ washout or in CytD-treated cells. These data were validated by analysis of the level of internal β1 integrin after PQ-induced endocytosis by quenching the cell surface fluorescence. Blocking the recycling with PQ increased the total level of endocytosed inactive β1 integrin almost twofold, whereas the difference was not significant for active β1 integrin (Figure 8C). PQ washout restored the levels of both conformations, but inhibition of actin polymerization during PQ washout clearly inhibited the recycling of inactive β1 integrin. CytD had no effect on active β1 integrin recycling. This suggests that the recycling of the inactive β1 integrin is an F-actin-dependent process.

### Inactive β1 integrin localizes to ARF 6-positive protrusions and endosomes

**Figure 9:**
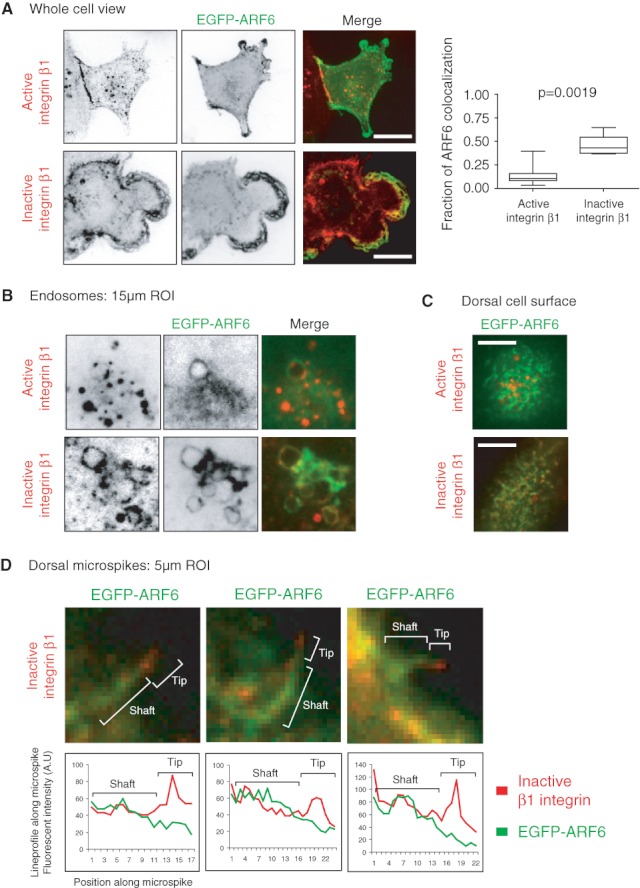
Inactive β1 integrin localizes to ARF6-positive endosomes and protrusions. MDA-MB-231 cells were transfected with EGFP-ARF6. Cells were surface labelled against inactive (mAb13) or active (12G10) β1 integrin. Integrins were allowed to endocytose 120 min, cells were fixed, counterstained and analysed under confocal microscope. A) Mid-sections are shown. Scale bar 10 µm. The fraction of ARF6 colocalization with active and inactive β1 integrin was quantified. Graph shows mean ± standard error of the mean. p Value was calculated using Mann–Whitney test. B) Fifteen micrometre ROIs of ARF6-positive endosomes are shown. C) Dorsal cell surfaces are shown. Scale bar 10 µm. D) Five micrometre ROIs of dorsal cell surface are shown with linescan along the ARF6-positive microspike. Shaft and tip regions are illustrated over the ROI and the corresponding region marked over the linescan.

ARF6 has been shown to localize to the plasma membrane and to regulate endosomal β1 integrin recycling and actin polymerization 21,44,45. In line with our data on the inactive β1 integrin recycling being more dependent on actin polymerization, we also see increased inactive β1 colocalization with ARF6 in protrusions, endosomes and in dorsal cell surface microspikes (Figure 9A–C). Active β1 integrin was found in the regions of ARF6 endosomes, but it did not localize to the limiting edges of ARF6 endosomes like the inactive β1 integrin did (Figure 9B). Also, active β1 integrin was not found in ARF6 protrusions and it did not colocalize in ARF6-positive dorsal microspikes (Figure 9A,C). Closer observation of ARF6-positive microspikes shows that the inactive β1 integrin localizes to the tips of the microspikes (Figure 9Dand Movie S1). Interestingly, these microspikes resemble greatly the actin tail comets pushing endosomes using actin polymerization for propulsion, as described by aunton et al 46.

**Figure 10:**
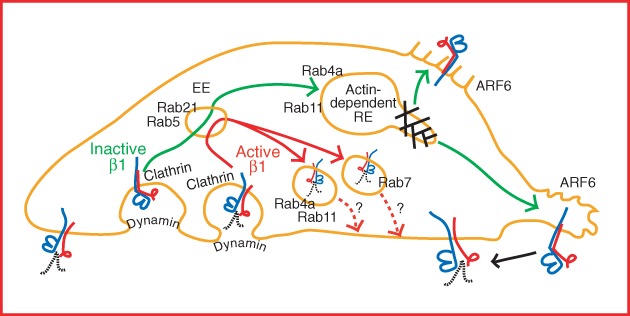
Model of distinct recycling routes of active and inactive β1 integrins. A model of active and inactive β1 integrin trafficking. Both conformations are endocytosed clathrin and dynamin dependently to early endosomes (EE) and to Rab5/Rab21-positive endosomes where the conformer separation probably takes place. The active β1 integrin is targeted to a Rab7 compartment, whereas the endocytosis of the inactive β1 integrin is balanced with rapid F-actin and Rab4-dependent recycling targeting integrins back to ARF6-positive protrusions on the plasma membrane. The active β1 integrin recycling is less efficient, and it is currently unclear whether ligand separation or integrin inactivation is needed as a signal for active integrin recycling and reusage. Active β1 integrin is seen in Rab4a- and Rab11-positive compartments, but more careful studies in respect to active β1 integrin recycling are needed. RE, recycling endosome.

Taken together, our data indicate that the inactive and active β1 integrins initially follow the same endocytosis routes and trafficking pathways but have distinct trafficking kinetics in cells (Figure 10). The active β1 integrin is targeted to a Rab7 compartment, whereas the endocytosis of the inactive β1 integrin is balanced with rapid F-actin and Rab4-dependent recycling targeting integrins back to ARF6-positive protrusions on the plasma membrane. In contrast, active β1 integrin has higher net endocytosis rate as it recycles with slower kinetics.

## Discussion

We have studied the endocytic trafficking of active and inactive β1 integrins. To the best of our knowledge, the data presented here include many previously undescribed details regarding integrin traffic. We show that integrins traffic with significantly different kinetics based on their activity status. The active receptor is predominantly localized to endosomes under steady-state conditions in untreated cancer cells. In contrast, the inactive integrin is predominantly localizing to detached membrane protrusions on the plasma membrane. The distinct localizations are due to different trafficking kinetics of the two receptor pools. We show that the inactive β1 integrin traffics through a Rab4- and actin-dependent fast-loop recycling pathway, whereas active integrin traffics predominantly along the previously described long-loop pathway 21. These data have important implications to our understanding of integrin function in cell migration as they demonstrate a clear link between the regulation of receptor activity and the dynamic regulation of the availability of the receptor on the plasma membrane RE, recycling endosome.

To date, limited data have been available on whether integrin traffic is regulated differently based on the activity of the receptor. Here, we describe a detection method for the different β1 integrin pools and conformational epitopes that is based on antibody labelling of cell surface β1 integrins. One of the major advantages of this antibody-based trafficking assay is the ability to distinguish between active and inactive β1 integrin pools and visualize the subcellular trafficking without the need for harsh acid-stripping regimens.

Here, we demonstrate that the net endocytic rate of the active β1 integrin is higher when compared against the inactive β1 integrin. Different recycling rates of endocytosed integrins have been attributed to changes in cell migration and invasion, co-trafficking of integrins and the EGFR to the plasma membrane and induced targeting of integrins to the plasma membrane in response to hypoxia 47. Here, we present evidence that the recycling of inactive and active β1 integrins display distinct kinetics and that the recycling of the inactive integrin is more dependent on actin. We describe a conformer-specific inactive β1 integrin-positive endosomal compartment that appears *de novo* after brief inhibition of receptor traffic with PQ. These compartments are positive for F-actin, and the recycling of inactive β1 integrin after PQ washout is dependent on actin polymerization. Owing to the faster recycling, the net endocytic rates of the inactive receptor appear lower. Interestingly, we show that the inactive integrin is rapidly returned to ARF6-positive membrane ruffles and dorsal cell surface microspikes. This would be in line with the possibility that inactive integrins are preferentially targeted to actively protruding areas of the plasma membrane where they would be available to be activated and incorporated into newly forming adhesion sites (Figure 10 ). However, this remains to be shown.

The endocytosis of both β1 integrin conformations was dynamin and clathrin dependent. However, we cannot fully exclude the possibility of other endocytic routes. In our system, caveolin-1 inhibition had no effect on the endocytosis. However, the role of caveolin-1 in β1 integrin endocytosis is most likely cell line specific as other studies clearly show that caveolin-1 is important in the endocytosis of β1 integrins 16. Interestingly, our results show caveolin-1 localizing to the rear of the motile MDA-MB-231 cells with no colocalization with the endocytosed β1 integrins. In contrast, colocalization is seen with clathrin and endocytosed active and inactive β1 integrins (Figure S5B,C). These observations are in line with previous studies where endocytic trafficking is shown to be toward the leading lamella and caveolar endocytosis would take place in the rear 1. Also, the observed requirement of dynamin-mediated abscission implies that the antibodies are not internalized via macropinocytosis.

The generally accepted view of β1 integrin traffic is that receptors are endocytosed via different routes and traffic via a Rab5/Rab21-positive early endosomal compartment *en route* to the Rab11/ARF6-positive recycling endosomes^48^,^49^. We see both conformations clearly colocalize with Rab5, Rab21, Rab4a and later to some extent with Rab11. Exit of β1 integrins from the early endosomes is dependent on a transient interaction between integrin α-tail and p120RasGAP which serves to displace Rab21 bound to the integrin α-tail 50. We find that under basal conditions, the limited number of inactive β1 vesicles do not overlap with the active integrin. However, once the recycling of the inactive β1 integrin is blocked, both conformations are detected in the same early endosomal compartment near the plasma membrane. This indicates that both conformations traffic through the early endosomes and that the inactive integrin is diverted to a fast-recycling pathway through a yet unidentified mechanism. Supporting this, the dominant-negative Rab4a inhibits the recycling of inactive β1 integrin but not the active β1 integrin. The active receptors traffic with slower kinetics, possibly because of the requirement for the ligand to be dissociated prior to receptor recycling. This is supported by a higher fraction of active β1 integrin found in Rab7-positive compartments.

In conclusion, we have developed a novel method to study β1 integrin trafficking. Using this method, we have identified differences in the trafficking of active and inactive β1 integrins. In the future, it will be interesting to study integrin trafficking from the point of view of the α-heterodimer since it has been suggested that the α-chains would have a role in integrin endocytic cycling 51.

## Materials and Methods

### Cell culture, reagents and DNA constructs

Human adenocarcinoma MDA-MB-231 cells were grown in Dulbecco's modified Eagle's medium supplemented with 10% foetal bovine serum (FBS), 1% l-glutamine and 1% non-essential amino acids. Human prostate cancer PC-3 cells were grown in RPMI-1640 medium supplemented with 10% FBS and 1% l-glutamine. Human NCI-H460 non-small cell lung cancer cells were grown in RPMI-1640, 10% FBS, 1% l-glutamine, 1% HEPES and 1% sodium pyruvate. Antibodies used in this study included: mouse anti-CD29 clone K20 (Beckman Coultier), mouse anti-CD29 MCA2028 clone 12G10 (Abcam), rat anti-CD29 mAb13 (BD Biosciences), mouse anti-CD29 clone 4B4 (Beckman Coultier), rat anti-CD29 9EG7 (BD Pharmingen), mouse anti-CD29 Huts-21 (BD Pharmingen), mouse anti-CD49b P1H5 (Santa Cruz), mouse anti-CD49b mab1998 (Millipore) and rabbit anti-Alexa Fluor 488 (Molecular Probes). Antibodies were labelled using APEX Alexa Fluor 488 antibody labelling and Alexa Fluor 488 protein labelling kits according to manufacturer's instructions (Molecular probes). His-tagged fibronectin fragment type III (7-10) was also labelled as above. Fluorescently labelled transferring (Alexa Fluor 570) was from Molecular Probes. pEGFP-C1 (Clontech), GFP-dynamin-2 K44A, GFP-Eps15 EH29 and GFP-caveolin-1 have been previously described 40–42. EGFP-tagged ARF6, Rab4a, Rab4a-S22N, Rab5, Rab7, Rab11, Rab21 and Rab25 have been previously described 10,13,52–56.

### Antibody-based internalization and recycling assay

Cells were plated on 96-well plates to 60–80% confluency. Plates were lifted on ice and allowed to cool down. Cell surface β1 integrins were labelled with indicated Alexa Fluor 488-conjugated antibodies in cell culture medium supplemented with 30 mmHEPES (pH 7.4) for 1 h on ice. Staining medium was aspirated, washed and replaced with fresh culture medium with 30 mm HEPES (pH 7.4). To start integrin internalization, 96-well plates were lifted on 37°C water bath and incubated for the indicated time-point. After internalization, plates were lifted on ice and cooled down. The fluorescence on cell surface was quenched by adding anti-Alexa Fluor 488 antibody and incubating for 1 h on ice. Cells were fixed in 4% paraformaldehyde (PFAH) for 20 min at room temperature, and internalized β1 integrin fluorescent intensities were measured using Acumen multiwell plate-reader (TTP LabTech). Fluorescence intensities were normalized against total surface staining per each antibody. To measure integrin recycling, cells were plated and cell surface β1 integrins were stained as above. Labelled β1 integrins were allowed to internalize for 30 min at 37°C. The surface was quenched as above. Cells were reincubated at 37°C water bath for the indicated time-points. After reincubation, the surface signal was requenched and cells were fixed. The fluorescent intensities were measured using Acumen multiwell plate-reader (TTP LabTech). Method outline is shown in Figure 3a.

### Biotin–IP-based integrin internalization and recycling assay. Western blot-and ELISA -based detection

Biotin-based assays were performed as described previously^10^,^13^. MDA-MB-231 or PC-3 cells were grown in 10% FBS-containing medium on 6 cm dishes to 80% confluence. The cells were placed on ice and washed once with cold PBS. Cell surface proteins were labelled with 0.5 mg/mL of EZ-link cleavable sulfo-NHS-SS-biotin (#21331; Thermo Scientific) in Hanks' balanced salt solution (H9269; Sigma) for 30 min at 4°C. Unbound biotin was washed away with cold medium, and prewarmed 10% serum-containing medium was added to cells. The biotin-labelled surface proteins were allowed to internalize at 37°C for the indicated time-points, after which the cells were placed quickly back on ice with cold medium. In the case of recycling, the cells were incubated at 37°C for 30 min to allow the internalization of all biotin-labelled integrins. The remaining biotin at the cell surface after internalization was removed with 60 mm MesNa (63705; sodium 2-mercaptoethanesulfonate: Fluka) in MesNa buffer (50 mm Tris–HCl, pH 8.6, 100 mm NaCl) for 30 min at 4°C, followed by quenching with 100 mm iodoacetamide (Sigma) for 15 min on ice. To detect the total amount of surface biotinylation, one of the cell dishes was left on ice after biotin labelling followed by treatment without reducing MesNa. Cells were washed with PBS and in the case of recycling, the cells were incubated again at 37°C for the indicated time-points followed by reduction and quenching of the recycled biotin-labelled surface molecules as above. The cells were lysed by scraping in lysis buffer [1.5% octylglucoside, 1% NP-40, 0.5% BSA, 1 mm EDTA with phosphatase and protease inhibitor coctails (Roche)] and incubation at 4°C for 20 min. Cell extracts were cleared by centrifugation (16 000 × *g*, 10 min, 4°C), and the biotinylated integrins were immunoprecipitated from the supernatant with 2 ng/μL of anti-β1 integrin antibody (CD29 K20; Custom Design Service, Beckman Coulter) and protein G sepharose beads (17-0618-01; GE Healthcare). Internalized integrins were detected from immunoblots with horseradish peroxidase (HRP)-conjugated anti-biotin antibody (#7075; Cell Signaling Technology), and after stripping the immunoblot, the total amount of immunoprecipitated integrins was detected with anti-β1 integrin antibody (MAB2252, Chemicon, or 610468, BD Transduction Laboratories). Enhanced chemiluminescence-detected biotin and β1 integrin signals were quantified as integrated densities of the protein bands with ImageJ (version 1.43u), and each biotin signal was normalized to the corresponding integrin signal. In the case of internalization, the relative amounts of biotin-labelled integrins inside the cell were normalized to the biotin signal of all surface labelled integrins, while in the case of recycling, the relative biotin signals were normalized to the corresponding biotin signal of all internalized integrins. The decreasing amount of biotin-labelled integrins inside the cell was inverted to represent the increase of β1 integrin recycling. The ELISA-based endocytosis assays were performed as described above. To determine the amount of internalized, biotinylated integrins, the cell lysate was incubated in a 96-well plate using the well-coating anti-β1 integrin antibody K20 and an HRP-coupled anti-biotin antibody for ELISA detection.

### Microscopy and image analysis

Cells were grown on Ibidi Ibitreat µ-Dishes or µ-slides (Integrated Biodiagnostics). Cells were fixed with 2% paraformaldehyde and 1 mm MgCl_2_ in PBS for 15 min at room temperature; the fixative was quenched with 50 mm NH_4_Cl in PBS and permeabilized with 0.2% Triton-X-100 in PBS for 15 min at room temperature. Samples were blocked and antibodies were diluted in 30% horse serum in PBS. Primary antibodies were used with predetermined optimal concentrations of 5–10 µg/mL. The concentration of Alexa-conjugated secondary antibodies (Invitrogen) was 5 µg/mL. Confocal 3D images were taken using Zeiss Axiovert 200M with spinning disc confocal unit Yokogawa CSU22 and Zeiss Plan-Neofluar 63xOil/1.4 NA objective. Z-stacks with 1 airy-unit (approximately 0.7 µm) optical slices were acquired with step size of 0.5 µm between slices. Image analysis and image projections were created with NIH ImageJ. JACop plugin was used to calculate colocalizations 57. β1-Integrin endocytosis was measured from mid-slice confocal images using ROI drawn inside the cell with plasma membrane as the boundary. Integrated densities were measured using ImageJ, and the inside signals were normalized against the total fluorescence signal of the same slice and cell (ROI around the whole cell).

### Flow cytometry

Cells were grown to 60–80% confluency and harvested with HyQTase (HyClone). Cells were diluted in culture medium with 30 mm HEPES (pH 7.4). Cells were lifted on ice, and cell surface β1 integrins were stained with primary antibody diluted 1:100 for 60 min at 4°C, followed by counterstaining with 1:400 diluted Alexa Fluor 488-conjugated secondary antibodies (Molecular Probes) for 60 min at 4°C. The internalization and recycling assays were performed as described. Cells were analysed using BD FACSCalibur and AccuriC6.

### Western blotting

Whole cell lysates were collected from nearly confluent six wells of a 6-well plate. Standard SDS sample buffer and scraping was used to lyse the cells. Standard western blotting techniques and Odyssey LICOR imaging system was used.

## Supporting Information

Figure S1 The quenching efficiency of anti‐Alexa Fluor 488 antibody and temperature dependency of the endocytosis. A) PC‐3 cells growing on 96‐wells were surface stained for 1 h on ice with Alexa Fluor 488‐labelled total β1 integrin antibody K20. After washing with cold growth medium, surface fluorescence was quenched with anti‐Alexa Fluor 488 antibody for 1 h on ice. Fluorescence was measured with a plate‐reader and normalized against non‐quenched cells. Graph shows mean ± standard error of the mean (SEM). B) PC‐3 cells (adherent and cells in suspension) were surface labelled with total β1 integrin antibody K20 for 1 h on ice. After washing with cold growth medium, cells were allowed to endocytose β1 integrin for 30 min at different temperatures on water bath. Graph shows mean ± SEM.

Figure S2 Comparison of the endocytosis and recycling assays. A) Comparison of total β1 integrin endocytosis in PC‐3 cells with K20 antibody using antibody and biotin–IP‐based assays. Graph shows mean ± standard error of the mean (SEM) of three independent experiments. Total surface and endocytosed biotin‐labelled β1 integrin are shown in the western blot. B) Endocytosis of total β1 integrin in MDA‐MB‐231 cells with K20 antibody measured with ELISA. Graph shows mean ± SEM of three independent experiments. C) Recycling of total β1 integrin in MDA‐MB‐231 cells with K20 antibody measured with ELISA. Graph shows mean ± SEM of three independent experiments.

Figure S3 The effects of anti‐β1 integrin antibodies on cell signalling. PC‐3 cells were surface stained with biotin or with monoclonal anti‐β1 integrin antibodies for 1 h on ice. The medium was changed and cells were incubated for 30 min at 37°C. Western blots of whole cell lysates were probed with (A) phospho‐FAK and (B) phospho‐p42/44 antibodies.

Figure S4 The endosomal trafficking pathway of active and inactive β1 integrins. MDA‐MB‐231 cells were transfected with EGFP‐tagged small Rab‐GTPases and surface stained with antibodies against active (12G10) (A) or inactive (mAb13) (B) β1 integrin. Integrins were allowed to endocytose for 120 min and cells were fixed, counterstained and analysed under confocal microscope. Mid‐slices and ROI are shown. Scale bar 10 µm.

Figure S5 Endocytosis routes of β1 integrins. A) MDA‐MB‐231 cells were transfected with GFP‐tagged dominant‐negative dynamin‐2 (K44A), dominant‐negative Eps15 (EH29), dominant‐negative caveolin‐1 (GFP‐caveolin‐1) and GFP alone. Cells were stained for 1 h on ice with fluorescently labelled transferrin (Alexa Fluor 570). Transferrin was allowed to endocytose 30 min and cells were fixed and analysed under confocal microscope. The level of endocytosed transferrin was quantified from confocal mid‐sections and normalized to the total staining of the cell using ROI drawn inside the cell versus ROI over the whole cell. Columns show mean ± standard error of the mean of GFP‐positive and GFP‐negative cells. p Values are calculated using Mann–Whitney test (*n* = 10). Scale bar 10 µm. B and C) MDA‐MB‐231 cells were surface labelled with anti‐active 12G10 (B) or anti‐inactive mAb13 (C) β1 integrin for 60 min on ice. Cells were washed with growth medium, and β1 integrin was allowed to endocytose for 30 min at 37°C. Cells were fixed, permeabilized and stained against caveolin‐1 (Cav‐1) and clathrin light chain (CLC). Confocal mid‐section of representative images is shown with ROI. Arrowheads show vesicles with colocalized clathrin and integrin β1 integrin. Scale bar 10 µm.

Figure S6 Inactive integrin β1 endosomes. NCI‐H460 cells were treated with 0.5 mm PQ for 30 min at 37°C and fixed, permeabilized and stained against inactive β1 integrin antibodies (mAb13, 1998, P1H5 and 4B4, respectively). Confocal mid‐section of representative cells with ROI is shown. Arrowheads point to endosomes.

Movie S1 Inactive β1 integrin is recycled to ARF6‐positive endosomes and membrane ruffles. Confocal stack showing MDA‐MB‐231 cell transfected with EGFP‐ARF6 (green). Cells were surface labelled with inactive β1 integrin (mAb13, red). One hundred and twenty minutes after endocytosis, cells were fixed, counterstained and analysed under confocal microscope. Confocal slices with 0.5 µm steps are shown. Bottom and dorsal sides are indicated.

## References

[1] Fletcher SJ, Rappoport JZ (2010). Moving forward: polarised trafficking in cell migration. Trends Cell Biol.

[2] Hynes RO (2002). Integrins: bidirectional, allosteric signaling machines. Cell.

[3] Luo BH, Carman CV, Springer TA (2007). Structural basis of integrin regulation and signaling. Annu Rev Immunol.

[4] Kim M, Carman CV, Springer TA (2003). Bidirectional transmembrane signaling by cytoplasmic domain separation in integrins. Science.

[5] Zaidel-Bar R, Itzkovitz S, Ma'ayan A, Iyengar R, Geiger B (2007). Functional atlas of the integrin adhesome. Nat Cell Biol.

[6] Sheetz MP, Felsenfeld DP, Galbraith CG (1998). Cell migration: regulation of force on extracellular-matrix-integrin complexes. Trends Cell Biol.

[7] Jiang G, Giannone G, Critchley DR, Fukumoto E, Sheetz MP (2003). Two-piconewton slip bond between fibronectin and the cytoskeleton depends on talin. Nature.

[8] Grant BD, Donaldson JG (2009). Pathways and mechanisms of endocytic recycling. Nat Rev Mol Cell Biol.

[9] Bretscher MS, Aguado-Velasco C (1998). Membrane traffic during cell locomotion. Curr Opin Cell Biol.

[10] Roberts M, Barry S, Woods A, van der Sluijs P, Norman J (2001). PDGF-regulated rab4-dependent recycling of alphavbeta3 integrin from early endosomes is necessary for cell adhesion and spreading. Curr Biol.

[11] Roberts MS, Woods AJ, Dale TC, Van Der Sluijs P, Norman JC (2004). Protein kinase B/Akt acts via glycogen synthase kinase 3 to regulate recycling of alpha v beta 3 and alpha 5 beta 1 integrins. Mol Cell Biol.

[12] Lobert VH, Brech A, Pedersen NM, Wesche J, Oppelt A, Malerod L, Stenmark H (2010). Ubiquitination of alpha 5 beta 1 integrin controls fibroblast migration through lysosomal degradation of fibronectin-integrin complexes. Dev Cell.

[13] Pellinen T, Arjonen A, Vuoriluoto K, Kallio K, Fransen JA, Ivaska J (2006). Small GTPase Rab21 regulates cell adhesion and controls endosomal traffic of beta1-integrins. J Cell Biol.

[14] Caswell P, Norman J (2008). Endocytic transport of integrins during cell migration and invasion. Trends Cell Biol.

[15] Upla P, Marjomaki V, Kankaanpaa P, Ivaska J, Hyypia T, Van Der Goot FG, Heino J (2004). Clustering induces a lateral redistribution of alpha 2 beta 1 integrin from membrane rafts to caveolae and subsequent protein kinase C-dependent internalization. Mol Biol Cell.

[16] Shi F, Sottile J (2008). Caveolin-1-dependent beta1 integrin endocytosis is a critical regulator of fibronectin turnover. J Cell Sci.

[17] Nishimura T, Kaibuchi K (2007). Numb controls integrin endocytosis for directional cell migration with aPKC and PAR-3. Dev Cell.

[18] Pellinen T, Tuomi S, Arjonen A, Wolf M, Edgren H, Meyer H, Grosse R, Kitzing T, Rantala JK, Kallioniemi O, Fassler R, Kallio M, Ivaska J (2008). Integrin trafficking regulated by Rab21 is necessary for cytokinesis. Dev Cell.

[19] Jones MC, Caswell PT, Norman JC (2006). Endocytic recycling pathways: emerging regulators of cell migration. Curr Opin Cell Biol.

[20] Fang Z, Takizawa N, Wilson KA, Smith TC, Delprato A, Davidson MW, Lambright DG, Luna EJ (2010). The membrane-associated protein, supervillin, accelerates F-actin-dependent rapid integrin recycling and cell motility. Traffic.

[21] Powelka AM, Sun J, Li J, Gao M, Shaw LM, Sonnenberg A, Hsu VW (2004). Stimulation-dependent recycling of integrin beta1 regulated by ARF6 and Rab11. Traffic.

[22] Puthenveedu MA, Lauffer B, Temkin P, Vistein R, Carlton P, Thorn K, Taunton J, Weiner OD, Parton RG, von Zastrow M (2010). Sequence-dependent sorting of recycling proteins by actin-stabilized endosomal microdomains. Cell.

[23] Palecek SP, Loftus JC, Ginsberg MH, Lauffenburger DA, Horwitz AF (1997). Integrin-ligand binding properties govern cell migration speed through cell-substratum adhesiveness. Nature.

[24] Tiwari S, Askari JA, Humphries MJ, Bulleid NJ (2011). Divalent cations regulate the folding and activation status of integrins during their intracellular trafficking. J Cell Sci.

[25] Tadokoro S, Shattil SJ, Eto K, Tai V, Liddington RC, de Pereda JM, Ginsberg MH, Calderwood DA (2003). Talin binding to integrin beta tails: a final common step in integrin activation. Science.

[26] Moser M, Nieswandt B, Ussar S, Pozgajova M, Fassler R (2008). Kindlin-3 is essential for integrin activation and platelet aggregation. Nat Med.

[27] Rantala JK, Pouwels J, Pellinen T, Veltel S, Laasola P, Mattila E, Potter CS, Duffy T, Sundberg JP, Kallioniemi O, Askari JA, Humphries MJ, Parsons M, Salmi M, Ivaska J (2011). SHARPIN is an endogenous inhibitor of beta1-integrin activation. Nat Cell Biol.

[28] Cluzel C, Saltel F, Lussi J, Paulhe F, Imhof BA, Wehrle-Haller B (2005). The mechanisms and dynamics of (alpha)v(beta)3 integrin clustering in living cells. J Cell Biol.

[29] Ezratty EJ, Bertaux C, Marcantonio EE, Gundersen GG (2009). Clathrin mediates integrin endocytosis for focal adhesion disassembly in migrating cells. J Cell Biol.

[30] Chao WT, Kunz J (2009). Focal adhesion disassembly requires clathrin-dependent endocytosis of integrins. FEBS Lett.

[31] Teckchandani A, Toida N, Goodchild J, Henderson C, Watts J, Wollscheid B, Cooper JA (2009). Quantitative proteomics identifies a Dab2/integrin module regulating cell migration. J Cell Biol.

[32] Valdembri D, Caswell PT, Anderson KI, Schwarz JP, Konig I, Astanina E, Caccavari F, Norman JC, Humphries MJ, Bussolino F, Serini G (2009). Neuropilin-1/GIPC1 signaling regulates alpha5beta1 integrin traffic and function in endothelial cells. PLoS Biol.

[33] Jokinen J, White DJ, Salmela M, Huhtala M, Kapyla J, Sipila K, Puranen JS, Nissinen L, Kankaanpaa P, Marjomaki V, Hyypia T, Johnson MS, Heino J (2010). Molecular mechanism of alpha2beta1 integrin interaction with human echovirus 1. EMBO J.

[34] Byron A, Humphries JD, Askari JA, Craig SE, Mould AP, Humphries MJ (2009). Anti-integrin monoclonal antibodies. J Cell Sci.

[35] Kozik P, Francis RW, Seaman MN, Robinson MS (2010). A screen for endocytic motifs. Traffic.

[36] Somasundaram B, Norman JC, Mahaut-Smith MP (1995). Primaquine, an inhibitor of vesicular transport, blocks the calcium-release-activated current in rat megakaryocytes. Biochem J.

[37] van Weert AW, Geuze HJ, Groothuis B, Stoorvogel W (2000). Primaquine interferes with membrane recycling from endosomes to the plasma membrane through a direct interaction with endosomes which does not involve neutralisation of endosomal pH nor osmotic swelling of endosomes. Eur J Cell Biol.

[38] Ng T, Shima D, Squire A, Bastiaens PI, Gschmeissner S, Humphries MJ, Parker PJ (1999). PKCalpha regulates beta1 integrin-dependent cell motility through association and control of integrin traffic. EMBO J.

[39] Stenmark H, Olkkonen VM (2001). The Rab GTPase family. Genome Biol.

[40] Altschuler Y, Barbas SM, Terlecky LJ, Tang K, Hardy S, Mostov KE, Schmid SL (1998). Redundant and distinct functions for dynamin-1 and dynamin-2 isoforms. J Cell Biol.

[41] Benmerah A, Bayrou M, Cerf-Bensussan N, Dautry-Varsat A (1999). Inhibition of clathrin-coated pit assembly by an Eps15 mutant. J Cell Sci.

[42] Pelkmans L, Kartenbeck J, Helenius A (2001). Caveolar endocytosis of simian virus 40 reveals a new two-step vesicular-transport pathway to the ER. Nat Cell Biol.

[43] Damke H, Baba T, Warnock DE, Schmid SL (1994). Induction of mutant dynamin specifically blocks endocytic coated vesicle formation. J Cell Biol.

[44] Radhakrishna H, Donaldson JG (1997). ADP-ribosylation factor 6 regulates a novel plasma membrane recycling pathway. J Cell Biol.

[45] Al-Awar O, Radhakrishna H, Powell NN, Donaldson JG (2000). Separation of membrane trafficking and actin remodeling functions of ARF6 with an effector domain mutant. Mol Cell Biol.

[46] Taunton J, Rowning BA, Coughlin ML, Wu M, Moon RT, Mitchison TJ, Larabell CA (2000). Actin-dependent propulsion of endosomes and lysosomes by recruitment of N-WASP. J Cell Biol.

[47] Pellinen T, Ivaska J (2006). Integrin traffic. J Cell Sci.

[48] Caswell PT, Norman JC (2006). Integrin trafficking and the control of cell migration. Traffic.

[49] Ivaska J, Heino J (2011). Cooperation between integrins and growth factor receptors in signaling and endocytosis. Annu Rev Cell Dev Biol.

[50] Mai A, Veltel S, Pellinen T, Padzik A, Coffey E, Marjomaki V, Ivaska J (2011). Competitive binding of Rab21 and p120RasGAP to integrins regulates receptor traffic and migration. J Cell Biol.

[51] Bretscher MS (1992). Circulating integrins: alpha 5 beta 1, alpha 6 beta 4 and Mac-1, but not alpha 3 beta 1, alpha 4 beta 1 or LFA-1. EMBO J.

[52] Gomes AQ, Ali BR, Ramalho JS, Godfrey RF, Barral DC, Hume AN, Seabra MC (2003). Membrane targeting of Rab GTPases is influenced by the prenylation motif. Mol Biol Cell.

[53] Aikawa Y, Martin TF (2003). ARF6 regulates a plasma membrane pool of phosphatidylinositol(4,5)bisphosphate required for regulated exocytosis. J Cell Biol.

[54] Lebrand C, Corti M, Goodson H, Cosson P, Cavalli V, Mayran N, Faure J, Gruenberg J (2002). Late endosome motility depends on lipids via the small GTPase Rab7. EMBO J.

[55] Wilcke M, Johannes L, Galli T, Mayau V, Goud B, Salamero J (2000). Rab11 regulates the compartmentalization of early endosomes required for efficient transport from early endosomes to the trans-golgi network. J Cell Biol.

[56] Caswell PT, Spence HJ, Parsons M, White DP, Clark K, Cheng KW, Mills GB, Humphries MJ, Messent AJ, Anderson KI, McCaffrey MW, Ozanne BW, Norman JC (2007). Rab25 associates with alpha5beta1 integrin to promote invasive migration in 3D microenvironments. Dev Cell.

[57] Bolte S, Cordelieres FP (2006). A guided tour into subcellular colocalization analysis in light microscopy. J Microsc.

