# Supplementary material for: Distinct Recycling of Active and Inactive β1 Integrins
Source: Traffic. 2012 Jan 31;13(4):610–25. doi: 10.1111/j.1600-0854.2012.01327.x (PMC3531618; doi:10.1111/j.1600-0854.2012.01327.x)
Supplement: Figure S1 — The quenching efficiency of anti‐Alexa Fluor 488 antibody and temperature dependency of the endocytosis. A) PC‐3 cells growing on 96‐wells were surface stained for 1 h on ice with Alexa Fluor 488‐labelled total β1 integrin antibody K20. After washing with cold growth medium, surface fluorescence was quenched with anti‐Alexa Fluor 488 antibody for 1 h on ice. Fluorescence was measured with a plate‐reader and normalized against non‐quenched cells. Graph shows mean ± standard error of the mean (SEM). B) PC‐3 cells (adherent and cells in suspension) were surface labelled with total β1 integrin antibody K20 for 1 h on ice. After washing with cold growth medium, cells were allowed to endocytose β1 integrin for 30 min at different temperatures on water bath. Graph shows mean ± SEM. [file tra0013-0610-SD1.doc]

**Distinct recycling of active and inactive β1 integrins**

Antti Arjonen^1,2,3^, Jonna Alanko^1,2,3^, Stefan Veltel^1,2,3^, and Johanna Ivaska^1,2,3, §^

**Supplemental Information**

**
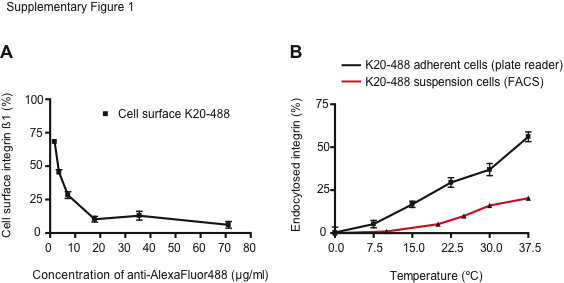
**

**Supplementary Figure 1. The quenching efficiency of anti-Alexa Fluor 488 antibody and temperature dependency of the endocytosis**

A) PC-3 cells growing on 96-wells were surface stained for 1h on ice with Alexa Fluor 488 -labelled total β1 integrin antibody K20. After washing with cold growth medium, surface fluorescence was quenched with anti-Alexa Fluor 488 antibody for 1 hour on ice. Fluorescence was measured with plate reader and normalized against non-quenched cells. Graph shows mean±SEM. B) PC-3 cells (adherent and cells in suspension) were surface labelled with total β1 integrin antibody K20 for 1 hour on ice. After washing with cold growth medium cells were allowed to endocytose β1 integrin for 30 minutes at different temperatures on water bath. Graph shows mean±SEM.
