# Supplementary material for: Distinct Recycling of Active and Inactive β1 Integrins
Source: Traffic. 2012 Jan 31;13(4):610–25. doi: 10.1111/j.1600-0854.2012.01327.x (PMC3531618; doi:10.1111/j.1600-0854.2012.01327.x)
Supplement: Figure S2 — Comparison of the endocytosis and recycling assays. A) Comparison of total β1 integrin endocytosis in PC‐3 cells with K20 antibody using antibody and biotin–IP‐based assays. Graph shows mean ± standard error of the mean (SEM) of three independent experiments. Total surface and endocytosed biotin‐labelled β1 integrin are shown in the western blot. B) Endocytosis of total β1 integrin in MDA‐MB‐231 cells with K20 antibody measured with ELISA. Graph shows mean ± SEM of three independent experiments. C) Recycling of total β1 integrin in MDA‐MB‐231 cells with K20 antibody measured with ELISA. Graph shows mean ± SEM of three independent experiments. [file tra0013-0610-SD2.doc]

**
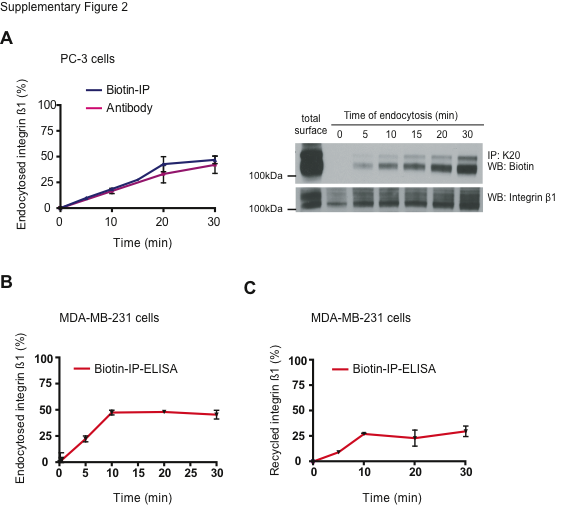
**

**Supplementary Figure 2. Comparison of the endocytosis and recycling assays**

A) Comparison of total β1 integrin endocytosis in PC-3 cells with K20 antibody using antibody and Biotin-IP based assays. Graph shows mean±SEM of three independent experiments. Total surface and endocytosed biotin-labelled β1 integrin are shown in the western blot. B) Endocytosis of total β1 integrin in MDA-MB-231 cells with K20 antibody measured with ELISA. Graph shows mean±SEM of three independent experiments. C) Recycling of total β1 integrin in MDA-MB-231 cells with K20 antibody measured with ELISA. Graph shows mean±SEM of three independent experiments.
