# Supplementary material for: Distinct Recycling of Active and Inactive β1 Integrins
Source: Traffic. 2012 Jan 31;13(4):610–25. doi: 10.1111/j.1600-0854.2012.01327.x (PMC3531618; doi:10.1111/j.1600-0854.2012.01327.x)
Supplement: Figure S3 — The effects of anti‐β1 integrin antibodies on cell signalling. PC‐3 cells were surface stained with biotin or with monoclonal anti‐β1 integrin antibodies for 1 h on ice. The medium was changed and cells were incubated for 30 min at 37°C. Western blots of whole cell lysates were probed with (A) phospho‐FAK and (B) phospho‐p42/44 antibodies. [file tra0013-0610-SD3.doc]

**
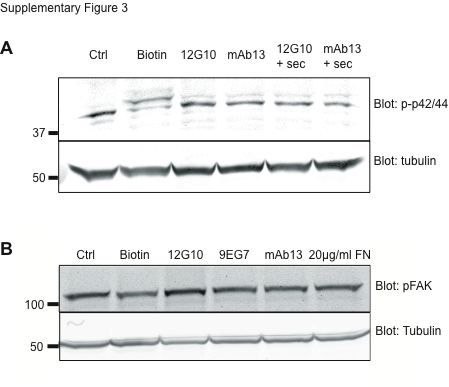
**

**Supplementary Figure 3. The effects of anti-β1 integrin antibodies to cell signalling**

PC-3 cells were surface stained with biotin or with monoclonal anti-β1 integrin antibodies for 1 hour on ice. The medium was changed and cells were incubated 30 minutes at 37˚C. Western blots of whole cell lysates where probed with A) phospho-FAK and B) phospho-p42/44 antibodies.
