# Supplementary material for: Distinct Recycling of Active and Inactive β1 Integrins
Source: Traffic. 2012 Jan 31;13(4):610–25. doi: 10.1111/j.1600-0854.2012.01327.x (PMC3531618; doi:10.1111/j.1600-0854.2012.01327.x)
Supplement: Figure S4 — The endosomal trafficking pathway of active and inactive β1 integrins. MDA‐MB‐231 cells were transfected with EGFP‐tagged small Rab‐GTPases and surface stained with antibodies against active (12G10) (A) or inactive (mAb13) (B) β1 integrin. Integrins were allowed to endocytose for 120 min and cells were fixed, counterstained and analysed under confocal microscope. Mid‐slices and ROI are shown. Scale bar 10 µm. [file tra0013-0610-SD4.doc]

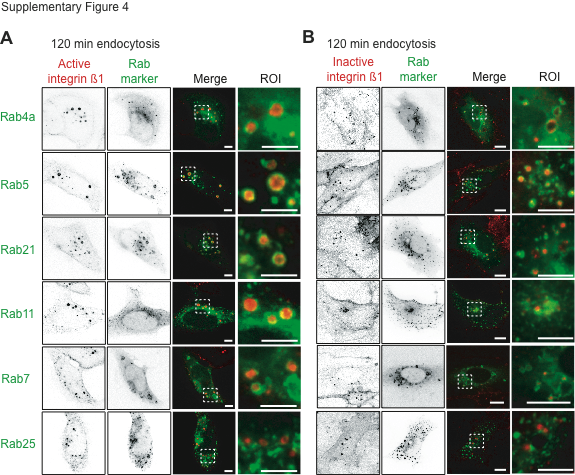


**Supplementary Figure 4. The endosomal trafficking pathway of active and inactive β1 integrins**

MDA-MB-231 cells were transfected with EGFP-tagged small Rab GTPases and surface stained with antibodies against active (12G10) (A) or inactive (mAb13) (B) β1 integrin. Integrins were allowed to endocytose for 120 minutes and cells were fixed, counterstained and analysed under confocal microscope. Mid-slices and ROI are shown. Scale bar 10µm.
