# Supplementary material for: Distinct Recycling of Active and Inactive β1 Integrins
Source: Traffic. 2012 Jan 31;13(4):610–25. doi: 10.1111/j.1600-0854.2012.01327.x (PMC3531618; doi:10.1111/j.1600-0854.2012.01327.x)
Supplement: Figure S5 — Endocytosis routes of β1 integrins. A) MDA‐MB‐231 cells were transfected with GFP‐tagged dominant‐negative dynamin‐2 (K44A), dominant‐negative Eps15 (EH29), dominant‐negative caveolin‐1 (GFP‐caveolin‐1) and GFP alone. Cells were stained for 1 h on ice with fluorescently labelled transferrin (Alexa Fluor 570). Transferrin was allowed to endocytose 30 min and cells were fixed and analysed under confocal microscope. The level of endocytosed transferrin was quantified from confocal mid‐sections and normalized to the total staining of the cell using ROI drawn inside the cell versus ROI over the whole cell. Columns show mean ± standard error of the mean of GFP‐positive and GFP‐negative cells. p Values are calculated using Mann–Whitney test (n = 10). Scale bar 10 µm. B and C) MDA‐MB‐231 cells were surface labelled with anti‐active 12G10 (B) or anti‐inactive mAb13 (C) β1 integrin for 60 min on ice. Cells were washed with growth medium, and β1 integrin was allowed to endocytose for 30 min at 37°C. Cells were fixed, permeabilized and stained against caveolin‐1 (Cav‐1) and clathrin light chain (CLC). Confocal mid‐section of representative images is shown with ROI. Arrowheads show vesicles with colocalized clathrin and integrin β1 integrin. Scale bar 10 µm. [file tra0013-0610-SD5.doc]

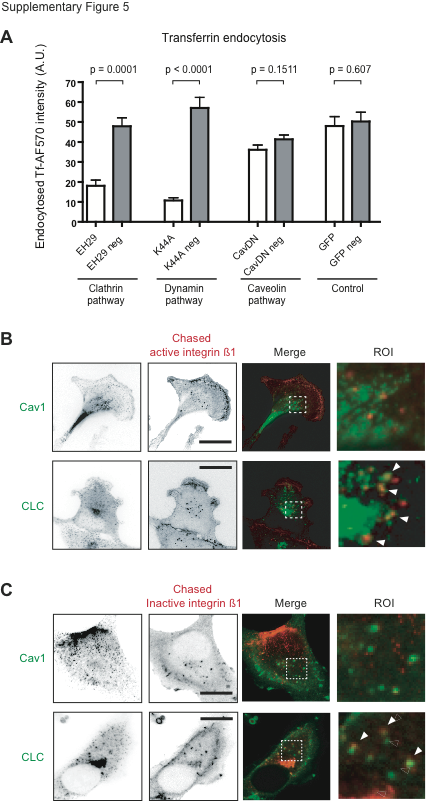


**Supplementary Figure 5. Endocytosis routes of β1 integrins**

1. MDA-MB-231 cells were transfected with GFP-tagged dominant negative dynamin-2 (K44A), dominant negative Eps15 (EH29), dominant negative caveolin-1 (GFP-Caveolin1) and GFP alone. Cells were stained 1h on ice with fluorescently labelled Transferrin (Alexa Fluor 570). Transferrin was allowed to endocytose 30 minutes and cells were fixed and analyzed under confocal microscope. The level of endocytosed transferrin was quantified from confocal mid-sections and normalized to the total staining of the cell using ROI drawn inside the cell versus ROI over the whole cell. Columns show mean+SEM of GFP-positive and GFP-negative cells. P-values are calculated using Mann Whitney test (n=10). Scale bar 10µm. B and C) MDA-MB-231 cells were surface labelled with anti-active 12G10 (B) or anti-inactive mAb13 (C) β1 integrin for 60 minutes on ice. Cells were washed with growth medium and β1 integrin was allowed to endocytose for 30 minutes at 37˚C. Cells were fixed, permiabilized and stained against caveolin-1 (Cav-1) and clathrin light chain (CLC). Confocal mid section of representative images is shown with ROI. Arrowheads show vesicles with colocalized clathrin and integrin β1 integrin. Scale bar 10µm.
