# Supplementary material for: Distinct Recycling of Active and Inactive β1 Integrins
Source: Traffic. 2012 Jan 31;13(4):610–25. doi: 10.1111/j.1600-0854.2012.01327.x (PMC3531618; doi:10.1111/j.1600-0854.2012.01327.x)
Supplement: Figure S6 — Inactive integrin β1 endosomes. NCI‐H460 cells were treated with 0.5 mm PQ for 30 min at 37°C and fixed, permeabilized and stained against inactive β1 integrin antibodies (mAb13, 1998, P1H5 and 4B4, respectively). Confocal mid‐section of representative cells with ROI is shown. Arrowheads point to endosomes. [file tra0013-0610-SD6.doc]

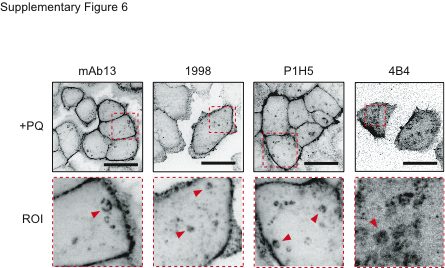


**Supplementary Figure 6. Inactive integrin β1 endosomes**

NCI-H460 cells were treated with 0.5 mM PQ for 30 minutes at 37˚C and fixed, permiabilized and stained against inactive β1 integrin antibodies (mAb13, 1998, P1H5 and 4B4, respectively). Confocal mid section is shown of representative cells with ROI. Arrowheads point to endosomes.
